# Idiopathic parotid pain: a rare clinical presentation of first bite syndrome without history of head and neck surgery or underlying malignancies

**DOI:** 10.1093/jscr/rjac566

**Published:** 2022-12-17

**Authors:** Matthew I Saleem, Sabreen Bhuiya, Tristan Tham, Alexandros Georgolios

**Affiliations:** Department of Otolaryngology, Donald and Barbara Zucker School of Medicine at Hofstra/Northwell, New York, NY 11549, USA; Department of Otolaryngology, Donald and Barbara Zucker School of Medicine at Hofstra/Northwell, New York, NY 11549, USA; Department of Otolaryngology, Donald and Barbara Zucker School of Medicine at Hofstra/Northwell, New York, NY 11549, USA; Poplar Bluff Regional Medical Center, Poplar Bluff, MO 63901, USA

## Abstract

First bite syndrome (FBS) has been previously characterised as a surgical complication, following head and neck surgical procedures. There are also rare reports in the literature associating FBS with malignancies of the head and neck. The term ‘idiopathic parotid pain’ (IPP) has been used recently to describe an exceedingly rare clinical presentation similar to FBS, but without history of head and neck surgery or malignancy. We present the rare case of a 65-year-old male diagnosed with IPP, our work-up and management.

## INTRODUCTION

First bite syndrome (FBS) has been previously characterized as a surgical complication, following procedures of the infratemporal fossa, parapharyngeal space, parotid glands and carotid endarterectomy [1–4]. There are also rare reports in the literature associating FBS with malignancies, specifically mucoepidermoid carcinoma of the parotid gland and adenoid cystic carcinoma of the submandibular gland [5, 6]. The clinical presentation is that of a sharp, unilateral preauricular or infra-auricular pain, experienced with the first bite of each meal. The pain is short lived and tends to diminish with subsequent bites until complete resolution [7].

The term ‘idiopathic parotid pain’ (IPP) has been used recently to describe a clinical presentation similar to FBS in Japanese patients with type 2 diabetes [8]. The difference between FBS and IPP is the absence of previous head and neck surgical procedures or underlying malignancies. The equivalent term ‘idiopathic first bite syndrome’, which has been used in the literature in rare case reports, is also associated with diabetes [9].

## CASE REPORT

A 65-year-old male is presented to the office, referred by his primary care physician, reporting severe, sharp, unilateral pain in the left pre-auricular area, associated with the first bite in every meal. The patient reports that the pain diminishes with every bite and completely resolves after the sixth or seventh bite. It happens every time in the initiation of his meals, regardless of the liquid or solid food quality. He reports significant discomfort with this condition, although he denies weight loss. This clinical presentation was gradually established over the last 2 years with continuous deterioration.

He is an insulin-dependent diabetic patient with overall good glycemic control, and is followed by his primary care physician. He has a history of hypertension and coronary artery disease. He is status post-coronary artery bypass grafting in 2015. He denies head and neck surgery, hemoptysis, odynophagia or voice changes. He has severe sensorineural hearing loss bilaterally, which is also longstanding. A computed tomography of the neck with contrast was ordered to rule out parotid disease, which was unremarkable.

**Figure 1:**
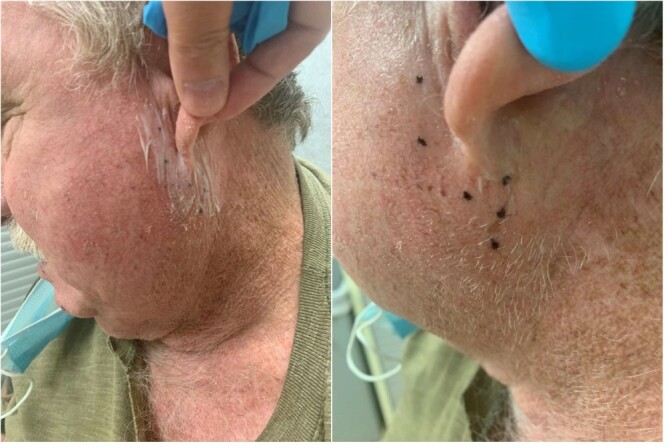
Topical anesthesia applied approximately 30 min prior to the procedure (left). Six injection points marked according to existing literature for post-surgical FBS (right).

The patient was treated with injection of botulinum toxin A to the unilateral parotid gland, according to the existing protocols used for post-operative FBS [10]. Specifically, we used 45–50 units of Botox in four areas of the parotid gland. The skin was prepped with topical anesthetic and the injections were performed approximately 30 min later to achieve topical analgesia as seen in Fig. 1. The patient was reassessed in 4 weeks after our intervention. His pre-intervention pain scale was reported 10/10, and his post-intervention pain scale 4 weeks after the injections was still 9/10. Four weeks after the intervention, the patient reported no subjective improvement as reflected on the pain scale. He stated that he had not noticed any improvement in the pain or the duration of his symptoms.

## DISCUSSION

FBS is a painful condition presenting as severe, sharp unilateral pain in the preauricular and infra-auricular region that is triggered by the first bite of a meal. The pain diminishes with subsequent bites until complete resolution. The pathophysiology of this syndrome is thought to be related to sympathetic denervation to the parotid gland [1, 11], and is known to be associated with previous head and neck surgical procedures and more rarely with underlying malignancies. The same clinical syndrome, when noticed without previous history of surgical procedures or malignancies, is reported as idiopathic FBS or IPP. In the present, we used the term IPP, in agreement with Chiba et al. [8] noting that the pathophysiology of idiopathic FBS is largely unknown and may be completely different than that of post-surgical FBS.

Only a handful number of cases of IPP have been reported in the literature, and are typically associated with diabetes [9]. Various treatments have been reported: stellate ganglion blocks [12], drug therapy with oxcarbazepine and gabapentine [13], pregabalin [9] and pharmacologic management with dietary modifications [8] have been used with various success rates. Botulinum toxin A injections to the parotid glands and submandibular glands have also been reported to successfully treat these rare cases [13]. Our additional case of a diabetic patient with IPP demonstrated negligible improvement in first bite pain with botulinum toxin A treatment. Previous reviews have supported the hypothesis that FBS is attributable to an imbalance between sympathetic and parasympathetic innervation of the parotid gland and is responsive to botulinum toxin A [14]. The fact that botulinum toxin A was ineffective in our patient further suggests that the pathophysiology of IPP is possibly distinct from post-surgical FBS and may be related to additional factors such as autoimmunity or inflammation.

We report our experience, with a 65-year-old male with a previous history of insulin-dependent diabetes who had a debilitating form of idiopathic FBS. The patient had a remote history of coronary artery bypass surgery, but no history of head and neck surgery or underlying malignancy. Botulinum toxin A injections to the parotid glands are commonly performed for different indications including sialorrhea, Frey’s syndrome and even post-surgical FBS [10, 15]. In our case, Botox failed to provide any improvement in the patient’s symptoms, which may reflect a different pathogenetic mechanism in idiopathic FBS versus the well-studied post-surgical FBS. That may also suggest a need for different dosing or repeat botulinum toxin injections. IPP is exceedingly rare and there are no established guidelines for its management; therefore, we arbitrarily used the injection technique and dosage previously described for the post-surgical FBS. We offered repeat Botox injections to the patient, but he declined and no further care was taken. The latter also illustrates the need for excellent pre-intervention communication with the patient regarding treatment expectations in these rare, understudied cases.

IPP is a complex pathology most likely distinct from post-surgical FBS. We report that botulinum toxin did not improve symptoms in this condition, suggesting further investigation into optimal management.
